# Association between serum methylmalonic acid and chronic kidney disease in adults: a cross-sectional study from NHANES 2013-2014

**DOI:** 10.3389/fendo.2024.1434299

**Published:** 2024-08-01

**Authors:** Zufa Zhang, Long Lv, Sheng Guan, Fengze Jiang, Danni He, Hongxuan Song, Weibing Sun, Sixiong Jiang, Feng Tian

**Affiliations:** ^1^ Department of Urology, Affiliated Zhongshan Hospital of Dalian University, Dalian, Liaoning, China; ^2^ Zhongshan Clinical Collage of Dalian University, Dalian, Liaoning, China; ^3^ Key Laboratory of Microenvironment Regulation and Immunotherapy of Urinary Tumors of Liaoning Province, Dalian, Liaoning, China

**Keywords:** serum methylmalonic acid, chronic kidney disease, NHANES, risk, association

## Abstract

**Introduction:**

Chronic kidney disease(CKD) is a global medical problem. Serum methylmalonic acid(MMA) is a serum marker associated with many diseases. This study aimed to investigate the association between MMA and CKD.

**Methods:**

Data from the National Health and Nutrition Examination Survey (NHANES) 2013-2014 were downloaded and analyzed. The association between MMA and CKD was confirmed by using multiple logistic regression modeling. The smooth curve fitting method was used to investigate the nonlinear relationship between them. Subgroup analyses and interaction tests were used to verify the stability of the association between different subgroups. Threshold effect analysis was used to determine the optimal control point for MMA.

**Results:**

There was a unique W-shaped nonlinear relationship between MMA and the risk of CKD, with a positive correlation between them (OR=1.66,95% CI:1.27, 2.17; P=0.0002). As the stage of CKD progressed, MMA levels increased. Age, hypertension, and serum vitamin B_12_ had significant influences on the association between MMA and the risk of CKD.

**Conclusion:**

Our findings revealed that serum MMA accumulation was positively associated with the risk of CKD. Serum MMA level may be a novel index to predict the development and course of CKD. This study may help in the early identification of people at risk for chronic kidney disease and provide new ideas and approaches for prevention and treatment.

## 1 Introduction

Chronic kidney disease (CKD) is a prevalent problem throughout the world, with its incidence on the rise, posing a growing threat to patients, the global economy, and even public health (1). CKD not only seriously affects the quality of life of patients, but also increases the risk of other chronic diseases, such as cardiovascular diseases and diabetes mellitus (2). Existing studies have shown that early symptoms of CKD are often non-specific and easily overlooked (3). Traditional renal function markers have several limitations and require further research and development (4). Therefore, it is crucial to accurately identify relevant markers in patients with potential CKD.

Methylmalonic acid(MMA) is a brief dicarboxylic acid that serves as a surrogate biomarker for mitochondrial dysfunction and oxidative stress. It has been demonstrated to disrupt specific inflammatory responses and contribute to redox stabilization. Meanwhile, MMA is recognized as a specific and sensitive marker for vitamin B_12_ deficiency (5–8). Several studies have reported ongoing updates and refinements in assays associated with serum MMA (9–11). The ability to test for serum MMA more efficiently and conveniently offers a range of clinical options. Serum MAA plays multiple roles in metabolic recoding and cellular signaling. It has been demonstrated that elevated MMA levels are associated with kidney disease and directly correlate with the risk of all-cause mortality (12, 13). Therefore, the association between serum MMA and the risk of CKD has potential value for clinical research.

However, there was a shortage of independent association studies on serum MMA and the risk of CKD. This study selected a nationally representative sample of adult patients to assess the association between MMA and the risk of CKD.

## 2 Methods

### 2.1 Study design and population

**Figure 1:**
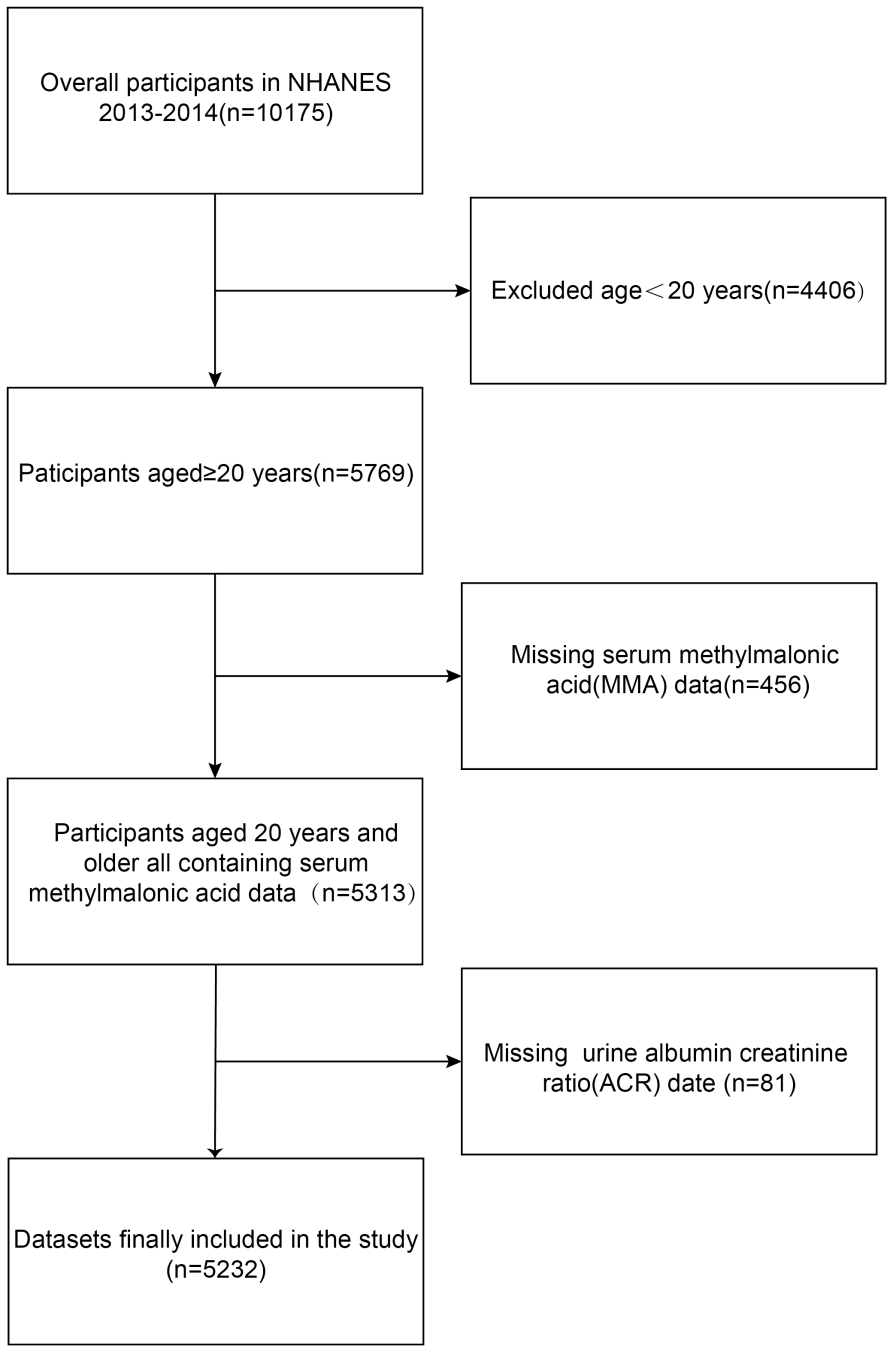
The flowchart of the inclusion-exclusion criteria.

The inclusion-exclusion criteria for the study are shown in **Figure 1**. This study was based on the 2013-2014 NHANES survey cycle because it included complete data on MMA concentrations and related covariates. Initially, the study recruited 10,175 participants and excluded 4,406 subjects younger than 20 years old, resulting in 5,769 participants being screened out. Additionally, 456 patients lacking complete MMA laboratory data and 81 without urine albumin creatinine ratio (ACR) data were excluded. Finally, 5,232 eligible subjects with complete MMA and ACR data, aged 20 years or older, were included in this study.

### 2.2 Serum methylmalonic acid

The exposure variable utilized in the study was serum MMA. The LC-MS/MS method was used to extract and analyze serum MMA. Before being sent to the National Center for Environmental Health for analysis, serum samples were prepared and stored at -30°C. Furthermore, the contract laboratory randomly duplicates 2% of all samples.

### 2.3 Chronic kidney disease

The CKD was defined as having an albumin-to-creatinine ratio (ACR) ≥ 30 or an estimated glomerular filtration rate (eGFR) <60. The Jaffe rate method was used to measure serum, plasma, or urine concentrations of creatinine. The solid-phase fluorescence immunoassay was used to determine urinary albumin. In calculating the glomerular filtration rate, this study employed the Chronic Kidney Disease Epidemiology Collaboration (CKD-EPI) formula (13). According to the KDOQI guideline introduction (14), CKD can be categorized into five stages based on eGFR and ACR: stage 1, (ACR ≥30 + eGFR≥90); stage 2,(ACR≥30 + eGFR 60-89); stage 3,(eGFR 30-59); stage 4(eGFR 15-29); stage 5,(eGFR<15). ACR is in mg/g and eGFR is in mL/min/l.73m^2^.

### 2.4 Covariates

Covariate data were collected through questionnaires, physical examinations, and laboratory tests. The following covariates were included: Gender, Age, Race and Ethnicity, Education level, Marital status, Physical activity, Smoking status, Alcohol, Body mass index (BMI) (kg/m2), Different ranges of vitamin B12(pg/mL), Diabetes, Hypertension, The ratio of family income to poverty(PIR), Alanine aminotransferase(ALT, U/L), Aspartate aminotransferase (AST, U/L), Total cholesterol (TC, mg/dL), Triglyceride(TG, mg/dL), Uric Acid (UA, mg/dL), Blood urea nitrogen (BUN, mg/dL). In NHANES, self-reported race and ethnicity information was derived from responses to race and Hispanic origin survey questions. Accordingly, we categorized participants into the following five races and ethnicities: non-Hispanic white, non-Hispanic black, other Hispanic, Mexican American, and other (including multiracial). Vitamin B_12_ is classified into low-level range, reference value range, and high-level range based on <300, 300-900, and ≥900. Educational attainment was measured by the questionnaire “What is the highest grade or level completed or degree earned?” for adults 20 years of age and older. Accordingly, we categorized educational attainment into three levels (less than high school, high school or GED, and above high school). Marital status was categorized into the following four groups: married, unmarried, living with a partner, and other (including widowed, divorced, or separated). Physical activity was collected through a physical activity questionnaire (PAQ-H) and was further categorized as vigorous, moderate, or less than moderate. Participants who answered “yes” to the following question were categorized as patients with a history of smoking, “Have you ever smoked at least 100 cigarettes in your life? 100 cigarettes in your life. “Had at least 12 alcohol drinks/1 yr?” If you answered yes, you were defined as a drinker. Those who answered “yes” and “borderline” to the question, “Have you ever been told by a doctor that you have diabetes, except during pregnancy?” were categorized as patients with a history of diabetes. The rest of the biochemical indicators are derived from the ‘Standard Biochemistry Profile’. During the physical examination, the height and weight of the subjects were recorded and BMI was subsequently calculated and categorized. BMI was categorized as <25, 25-30, and ≥30.

### 2.5 Statistical analysis

As recommended by the NHANES guidelines (14), we considered both complex sampling designs and sampling weights in the process of analyzing NHANES data. The concentration and distribution of MMA in serum were collected. Normally distributed continuous variables are expressed as mean ± SD, while skewed continuous variables are expressed as median (IQR). Categorical variables can be described by the count of observations in each category (absolute values) and their respective proportions (percentages). The association between MMA and the risk of CKD was assessed by multiple regression modeling, presenting odds ratios (OR) and 95% confidence intervals (CI). The dose-response relationship between them was further explored using Restricted Cubic Spline Curve (RCS), with subgroup analyses performed to observe their robustness. All statistical analyses were performed using Empower software(X&Y Solutions, Inc., Boston, MA, USA) and R3.4.3(The R Foundation, http://www.R-project.org). Descriptive statistics were descriptive for all participants. P < 0.05 was considered statistically significant.

## 3 Results

### 3.1 Participants and demographic baseline characteristics

Baseline demographic characteristics of eligible subjects are shown in **Table 1**. The study included a total of 5,232 adult patients with data on MMA and the risk of CKD, among whom 922 (17.62%) had CKD. The median MMA concentration for all subjects was 144.00 nmol/L (Q1 = 109.00, Q3 = 191.25). The median age of participants was 48 years, with 2,501 (47.80%) males and 2,731 (52.20%) females. Most of the participants were non-Hispanic whites (2,272,43.43%). At baseline, subjects in the highest quartile had a higher risk of CKD compared to the lowest quartiles (33.18%, P=0.001). They were mainly male, older, married, with diabetes mellitus, accompanied by drinking, smoking, and physical inactivity. In addition, participants in the highest quartile of serum MMA exhibited significantly higher levels of BUN, and UA compared to those in the lowest quartile, whereas TC and ALT levels were lower in the highest quartile than in the lowest quartile.

**Table 1:** Baseline characteristics of the study participants.

Characteristics	TotaTotal (n=5232)	Serum MMA levels, (nmol/L)				P value
		Quartile 1 < 109	Quartile 2 109-143	Quartile 3 143-191.25	Quartile 4 ≥191.25	
		(N=1281)	(N=1307)	(N=1336)	(N=1308)	
Gender, n (%)						0.0443
Male	2501(47.80)	43.22 (40.30,46.19)	50.56 (46.11,55.19)	50.37 (47.32,53.42)	48.04 (43.61,52.51)	
Female	2731(52.20)	56.78 (53.81,59.70)	49.35 (44.81,53.89)	49.63 (46.58,5268)	51.96 (47.49,56.39)	
Age, years	48(34,63)	40.05 (39.12,40.98)	44.06 (42.59,45.52)	48.53 (47.60,49.45)	55.61 (54.36,56.86)	<0.001
Race and ethnicity, n (%)						<0.001
Mexican American	711(13.59)	19.22 (13.72,26.24)	8.62 (5.50,13.26)	5.91 (3.71,9.29)	5.01 (2.86,8.64)	
Other Hispanic	467(8.93)	7.91 (5.68,10.91)	6.71 (4.15,10.68)	4.75 (2.88,7.73)	3.37 (2.15,5.24)	
Non-Hispanic White	2272(43.43)	42.46 (35.47,49.75)	65.17 (56.84,72.67)	74.83 (68.43,80.31)	78.22 (72.05,83.33)	
Non-Hispanic Black	1025(49.59)	18.78 (14.21,24.40)	11.54 (8.42,15.62)	8.15 (5.65,11.61)	6.43 (4.45,9.19)	
Other Races	757(14.47)	11.64 (9.64,13.97)	7.96 (6.14,10.25)	6.36 (5.24,7.71)	6.98 (4.94,9.78)	
PIR	2.12(1.12,3.92)	2.73 (2.56,2.89)	2.90 (2.68,3.13)	3.13 (2.93,3.33)	2.80 (2.58,3.01)	<0.001
Education level, n (%)						0.0046
Less than high school	2295 (43.86)	37.21 (32.22,42.49)	34.21 (28.10,40.90)	35.60 (31.26,40.19)	40.69 (35.18,46.44)	
High school or GED	1621(30.98)	36.81 (33.38,40.38)	33.84 (30.71,37.13)	31.83 (27.63,36.35)	29.44 (26.04,33.09)	
Above high school	1316 (25.15)	25.98 (22.05,30.33)	31.94 (27.31,36.96)	32.56 (27.14,38.50)	29.87 (25.21,34.99)	
Marital status, n (%)						<0.001
Married	2739(52.3)	50.75 (44.82,56.65)	56.82 (51.94,61.56)	60.73 (55.70,65.55)	54.08 (50.07,58.04)	
Never married	982(18.8)	25.44 (20.27,31.41)	19.98 (16.79,23.60)	16.80 (13.83,20.26)	12.63 (10.00,15.84)	
Living with a partner	382(7.3)	10.17 (7.42,13.80)	7.04 (5.48,9.01)	6.22 (4.15,9.23)	4.85 (3.46,6.77)	
Other	1129(21.6)	13.64 (11.63,15.93)	16.16 (13.91,18.70)	16.24 (14.52,18.12)	28.43 (23.83,33.53)	
Alcohol, n (%)						0.0171
Yes	3780 (72.25)	75.04 (70.63,78.99)	79.78 (73.38,84.95)	79.92 (73.70,84.97)	75.23 (69.64,80.09)	
No	1452 (27.75)	24.96 (21.01,29.37)	20.22 (15.05,26.62)	20.08 (15.03,26.30)	24.77 (19.91,30.36)	
Diabetes, n (%)						<0.001
Yes	642 (12.27)	7.78 (6.37,9.48)	8.37 (6.32,11.02)	8.83 (7.49,10.38)	14.18 (11.94,16.76)	
No	4590 (87.73)	92.22 (90.52,93.63)	91.63 (88.98,93.68)	91.17 (89.62,92.51)	85.82 (83.24,88.06)	
Hypertension, n(%)						<0.001
Yes	1939(37.06)	25.70 (22.40,29.30)	29.29 (25.90,32.91)	36.10 (32.13,40.27)	45.86 (43.28,48.45)	
No	3293(62.94)	74.30 (70.70,77.60)	70.71 (67.09,74.10)	63.90 (59.73,67.87)	54.14 (51.55,56.72)	
Smoking status, n(%)						<0.001
Yes	2268 (43.35)	37.18 (33.05,41.51)	42.90 (39.72,46.14)	44.45 (40.52,48.46)	47.71 (42.99,52.47)	
No	2964 (56.65)	62.82 (58.49,66.95)	57.10 (53.86,60.28)	55.55 (51.54,59.48)	52.29 (47.53,57.01)	
Physical activity, n(%)						<0.001
Vigorous	1154(22.06)	28.70 (25.61,32.00)	26.29 (21.57,31.62)	25.65 (21.98,29.69)	17.63 (14.33,21.50)	
Moderate	1448(27.68)	28.03 (24.26,32.13)	29.46 (25.78,33.44)	28.51 (25.38,31.87)	28.18 (24.52,32.14)	
Less than moderate	2630(50.26)	43.27 (38.80,47.86)	44.25 (39.46,49.14)	45.84 (42.99,48.72)	54.19 (48.89,59.41)	
BMI, n(%)						0.0640
<25	1560(29.82)	28.88 (26.04,31.89)	28.37 (25.65,31.27)	31.69 (28.34,35.24)	29.71 (26.00,33.70)	
25-30	1705(32.58)	29.56 (26.34,33.00	34.54 (31.34,37.88)	34.26 (30.50,38.22)	33.01 (30.31,35.82)	
≥30	1967(37.60)	41.56 (38.91,44.27)	37.09 (33.90,40.40)	34.05 (30.02,38.33)	37.29 (33.31,41.44)	
Vitamin B_12_, n(%)						<0.001
low-level range	224(4.28)	0.24 (0.08,0.69)	0.48 (0.17,1.36)	1.10 (0.70,1.72)	5.34 (3.51,8.04)	
reference value range	4524(86.47)	83.21 (79.99,86.00)	87.92 (85.75,89.90)	88.20 (85.97,90.13)	87.62 (84.66,90.08)	
high-level range	484(9.25)	16.55 (13.78,19.77)	11.60 (9.76,13.73)	10.69 (8.86,12.86)	7.04 (5.49,9.00)	
ALT, U/L	20(16,28)	26.61 (24.79,28.43)	25.91 (24.46,27.35)	25.22 (24.17,26.28)	23.85 (22.44,25.26)	0.0217
AST, U/L	22(19,27)	25.47 (23.59,27.36)	25.40 (24.44,26.36)	25.31 (24.52,26.11)	25.29 (24.22,26.37)	0.9973
BUN, mg/dL	12(10,16)	11.24 (10.94,11.54)	12.52 (12.12,12.92)	13.38 (13.12,13.64)	15.77 (15.19,16.34)	<0.001
TC, mg/dL	187(162,216)	190.77 (187.95,193.59)	189.40 (185.44,193.36)	192.91 (190.51,195.32)	191.20 (187.76,194.64)	0.5432
TG, mg/dL	120(80,187)	145.85 (129.17,162.53)	146.87 (137.25,156.48)	151.53 (143.50,159.56)	167.47 (158.66,176.29)	0.1031
UA, mg/dL	5.30 (4.40,6.30)	5.12 (5.05,5.19)	5.44 (5.35,5.54)	5.45 (5.35,5.54)	5.57 (5.42,5.73)	<0.001
CKD, n(%)						<0.001
Yes	922 (17.62)	9.77 (7.59,12.48)	8.59 (6.84,10.75)	13.84 (11.65,16.37)	28.71 (24.89,32.86)	
No	4310 (82.38)	90.23 (87.52,92.41)	91.41 (89.25,93.16)	86.16 (83.63,88.35)	71.29 (67.14,75.11)	

MMA, methylmalonic acid; CKD, chronic kidney disease; UA, uric acid; TG, triglyceride; TC, Total cholesterol; BUN, blood urea nitrogen; AST, aspartate aminotransferase; BMI, body mass index; PIR, the ratio of family income to poverty.

### 3.2 Association between serum MMA and the risk of having CKD

The study investigated the association between serum MMA and the risk of CKD by constructing a multivariate logistic regression model (**Table 2**). Serum MMA was converted from a continuous variable to a categorical variable for multivariate logistic regression analysis. Serum MMA was categorized by interquartile ranges. Models 1 and 2 presented a positive association between serum MMA and the risk of CKD in the highest quartile of participants. Specifically in the fully adjusted Model 3, this positive association persisted, albeit with slightly reduced strength compared to the previous models(OR=1.66,95% CI:1.27, 2.17; P=0.0002).

**Table 2:** Associations between serum MMA and CKD in the multiple regression mode.

Variable	ln MMA(nmol/L)	Quartiles of MMA levels			
		Q1	Q2	Q3	Q4
	OR(95%CI), P value	OR(95%CI), P value	OR(95%CI), P value	OR(95%CI), P value	OR(95%CI), P value
Model 1	3.45 (2.98, 3.99) <0.0001	Reference	1.09 (0.85, 1.40), 0.5072	1.77 (1.40, 2.23), <0.0001	4.47 (3.60, 5.55),<0.0001
Model 2	2.30 (1.97, 2.70) <0.0001	Reference	0.92 (0.71, 1.20),0.5282	1.24 (0.96, 1.59),0.0939	2.51 (1.98, 3.19),<0.0001
Model 3	1.70 (1.41, 2.07) <0.0001	Reference	0.78 (0.59, 1.03), 0.0752	1.05 (0.80, 1.36), 0.7301	1.66 (1.27, 2.17), 0.0002

Model 1: No adjustment for any covariates. Model 2: Adjusted for Gender, Age, Race and ethnicity. Model 3: Adjusted for Gender, Age, Race and ethnicity, PIR, Education level, Marital status, Alcohol, Diabetes, Hypertension, Smoking status, Physical activity, BMI, serum Vitamin B_12_, ALT, AST, BUN, TC, TG, UA. Abbreviation: OR, odds ratio; 95% CI, 95% confidence interval. MMA, methylmalonic acid; CKD, chronic kidney disease; UA, uric acid; TG, triglyceride; TC, Total cholesterol; BUN, blood urea nitrogen; AST, aspartate aminotransferase; BMI, body mass index; PIR, the ratio of family income to poverty.

### 3.3 Distribution and association of MMA in different CKD stages

**Figure 2:**
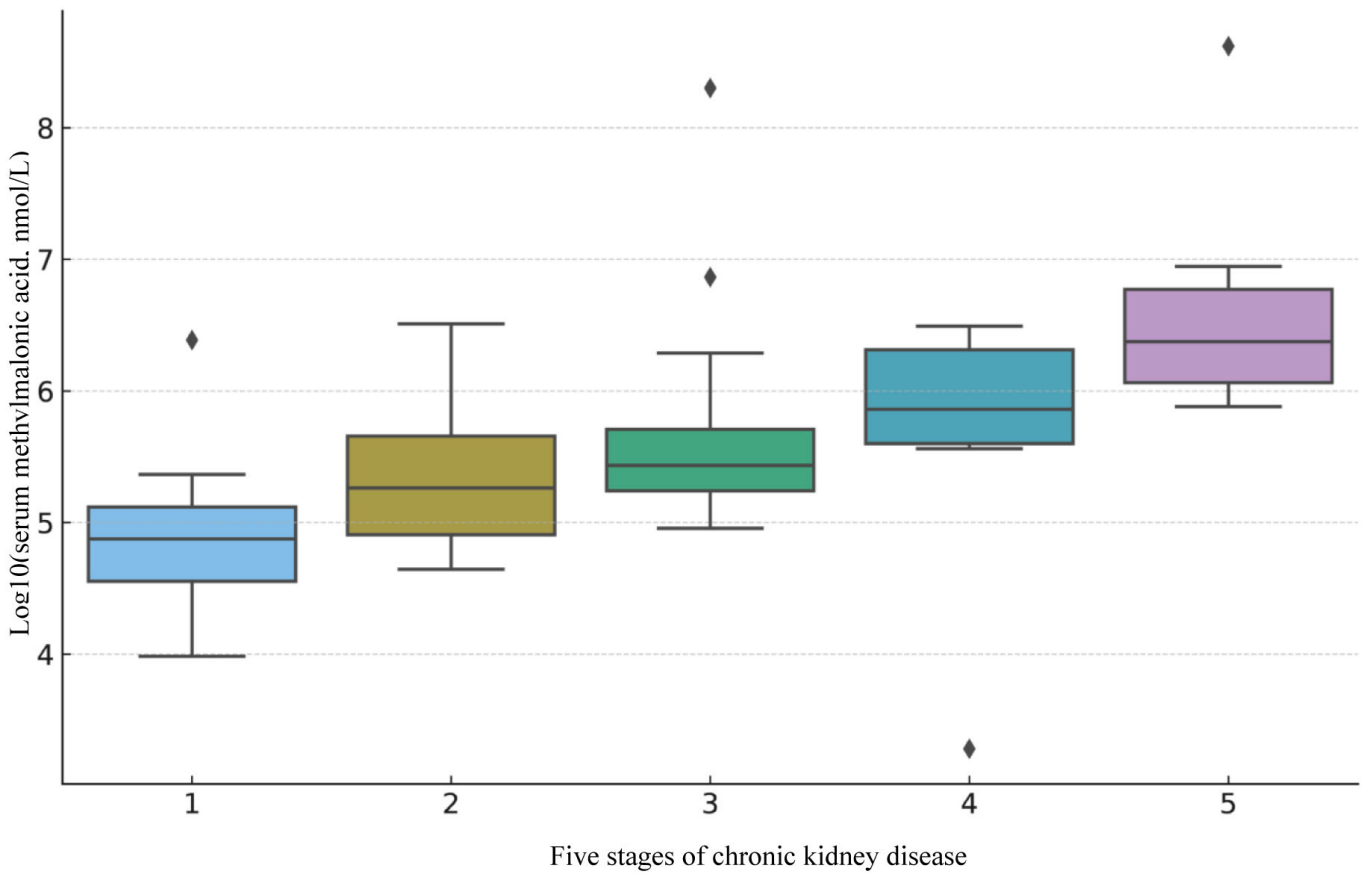
Distribution of MMA across CKD stages.

Eligible subjects underwent CKD staging to observe the distribution and association between MMA and different CKD stages. The results of a subsequent Kruskal-Wallis test (**Supplementary Material**) confirmed that the distribution of MMA among different CKD stages was significantly different (H=50.47, P=2.88e-10). To demonstrate a more intuitive visualization of their distribution and association, we visualized the data distributions of ln MMA across different stages of CKD(**Figure 2**). This study demonstrated a positive association between MMA and different stages of CKD. The median and range of distribution of MMA levels increased with increasing CKD stage, especially in more advanced CKD stages.

### 3.4 Subgroup analysis

**Figure 3:**
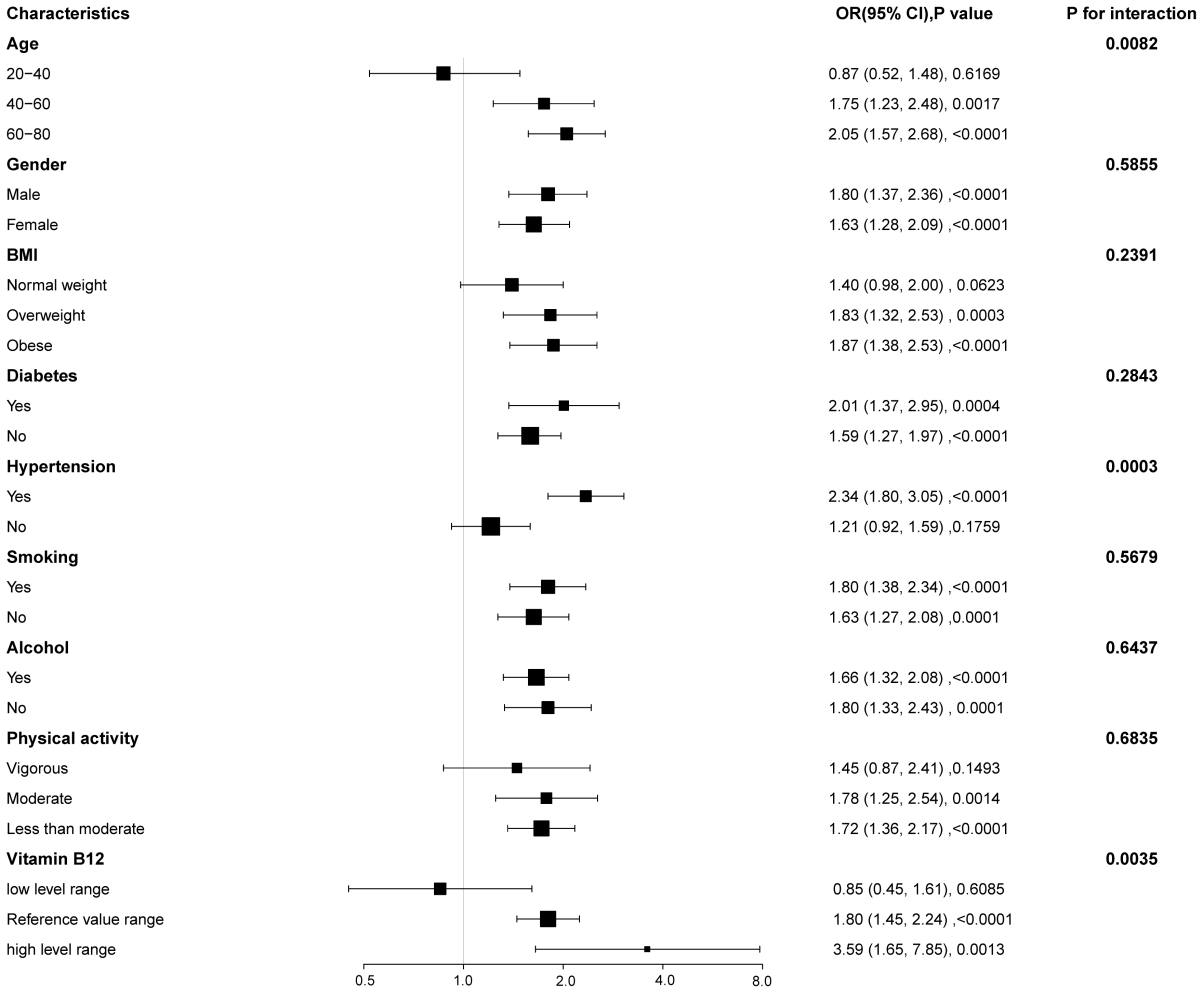
Forest plot for subgroup analysis of serum MMA and CKD.

To further explore whether there was consistency in the association between MMA and the risk of CKD across several subgroups, interaction and subgroup analyses were conducted (**Figure 3**). The results observed markedly association in gender, diabetes mellitus, smoking, and alcohol consumption subgroups (all P < 0.05). A higher risk of MMA association with CKD was shown in participants aged 40-80 years, overweight or obese, hypertensive, less than moderate, and with normal or high vitamin B_12_. In addition, the interaction test revealed that there was a significant interaction between MMA and the risk of CKD in terms of age, hypertension, and different levels of vitamin B_12_ (all P for interaction < 0.05). The results indicated these factors may play crucial roles as modifiers in the association between MMA and the risk of CKD.

### 3.5 Nonlinear dose-response relationship in the association between MMA and CKD

**Figure 4:**
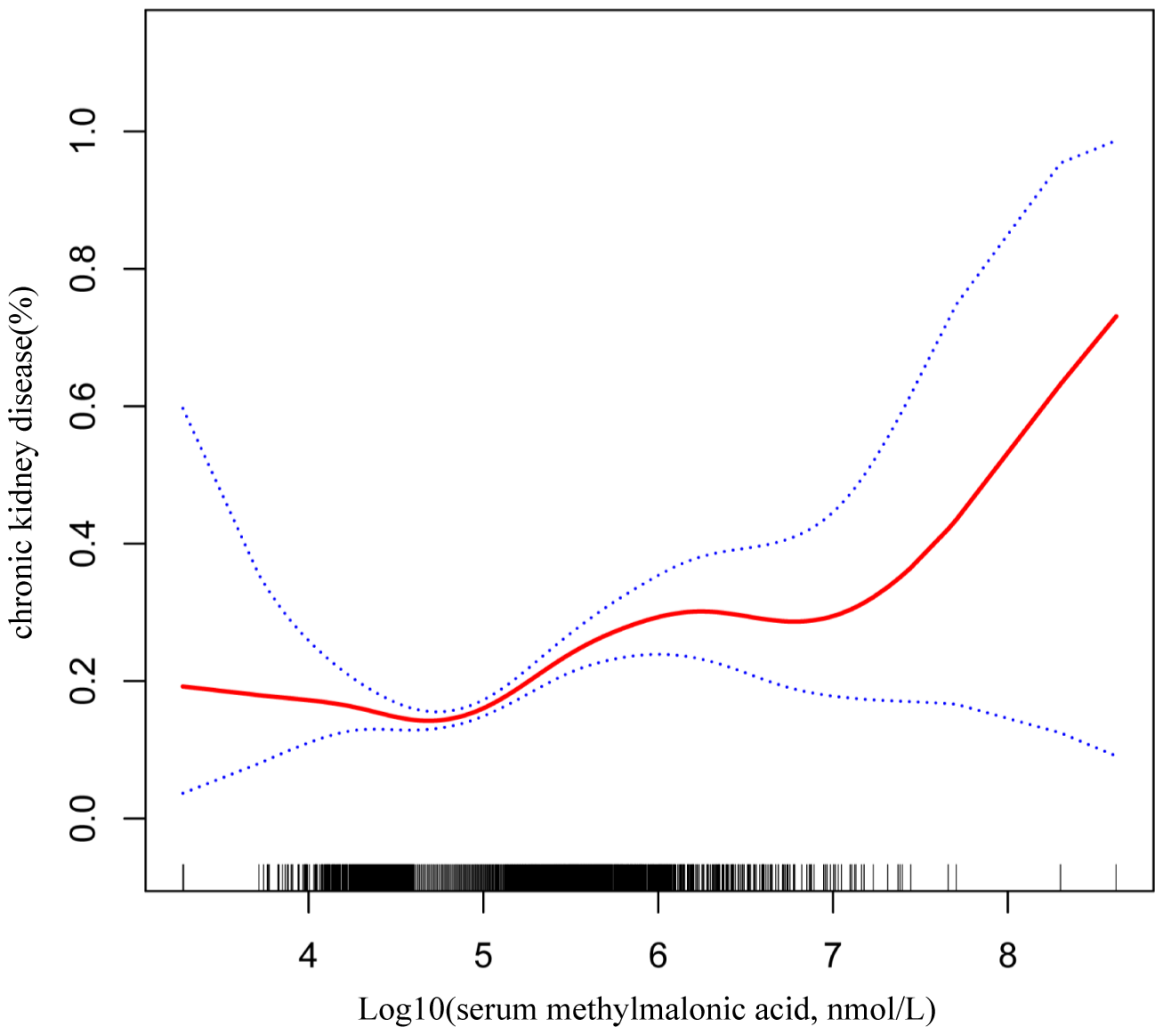
W-shaped restricted cubic spline plot of serum MMA and the risk of CKD. The solid red line represents the smooth curve fit between variables. Blue bands represent the 95% confidence interval from the fit.

**Figure 5:**
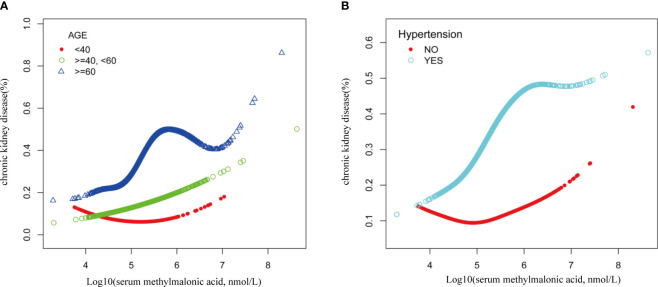
Nonlinear association of serum MMA with CKD risk by age **(A)** and hypertension **(B)** grouping.

Smooth curve fitting was employed to explore the nonlinear dose-response relationship between CKD and MMA (**Figure 4**). The result revealed a specific “W”-shaped nonlinear positive association between MMA and the risk of CKD (P < 0.05). The optimal control value (K) was estimated by threshold effect analysis. The results indicated that the association between MMA and the risk of CKD changed at approximately 4.77 (**Table 3**). Above the value of K, a strong positive association was observed between MMA and a higher risk of CKD (P<0.0001). In addition, based on the results of the interaction test, participants were stratified by age and hypertension status. Smoothed curve fitting was utilized to observe the difference in the nonlinear relationship between MMA and the risk of CKD in these two strata (**Figure 5**). When subgrouped by age, the risk of having CKD increased progressively with higher MMA concentrations in people aged 40-60 years. And there is a high-risk peak in people over 60 years of age. High levels of MMA in people with hypertension are more likely to lead to CKD.

**Table 3:** Threshold effect analysis for the association between MMA(ln transform) and CKD.

ln transform MMA (nmol/L)	OR(95%CI)	*P* value	value
< K slope 1	0.70 (0.36, 1.37)	0.2975	
≥ K slope 2	1.99 (1.59, 2.50)	<0.0001	
log-likelihood ratio test		0.009	
Inflection point(K)			4.77

CKD, chronic kidney disease; OR, odds ratio;95%CI, 95% confidence interval. Adjusted for Gender, Age, Race and ethnicity, PIR, Education level, Marital status, Alcohol, Diabetes, Hypertension, Smoking status, Physical activity, BMI, serum Vitamin B_12_, Alanine aminotransferase (ALT), Aspartate aminotransferase(AST), Blood urea nitrogen(BUN), Total serum cholesterol(TC), Triglyceride (TG), uric acid(UA).

## 4 Discussion

To our knowledge, this is the first study to independently assess the association between serum MMA and the risk of CKD. In this study, we recruited 5,232 eligible participants, of whom 922 (17.62%) had CKD. In the fully adjusted model, a nonlinear positive correlation was found between serum MMA and CKD, with CKD prevalence increasing across higher quartiles of serum MMA. It’s worth noting that the association is not unexpected since MMA is cleared by the kidney. Additionally, serum MMA levels increased with advancing CKD stages. Age, hypertension, and serum vitamin B_12_ may significantly influence the association between serum MMA and CKD.

It has been shown that correcting MMA for GFR assessed by creatinine enhances its utility as a marker of vitamin B_12_ deficiency (15). Some studies have also confirmed the limitations of traditional biomarkers of renal insufficiency, such as serum creatinine (16). Serum cystatin C has been recognized as an early and accurate biomarker of CKD. It is useful in patients where creatinine is not available as a marker or where glomerular filtration rate (GFR) measurements are complex (17). However, certain limitations have been confirmed in clinical practice (18). Therefore, other serologic markers potentially associated with CKD need to be further explored. While testing for serum markers can be a clinical practice, detecting serum MMA is not routine and may be more costly compared to standard blood tests. Nonetheless, its potential clinical applications remain promising in specific contexts.

MMA is an age-related byproduct of propionic acid metabolism that has been shown to promote tumorigenesis and progression (19, 20). The mechanism linking MMA and CKD is currently unknown. The key enzyme of MMA removal, methylmalonyl-CoA mutase, is dependent upon processed vitamin B_12_ (adenosyl cobalamin) and serum MMA can therefore serve as an indicator for assessing the metabolic status of vitamin B_12_ (21). Studies have shown that vitamin B_12_ deficiency is strongly associated with abnormally elevated serum MMA (22). Vitamin B_12_ deficiency can lead to hyperhomocysteinemia, which promotes kidney injury by inducing oxidative stress and inflammatory responses (23).

MMA is also involved in substance metabolism and cell signaling. The isotopic labeling method has revealed that the majority of MMA undergoes intracellular metabolism, forming numerous unknown products (24). Clinical case studies of individuals with small intestinal bacterial overgrowth corroborate the physiological low expression of MMA *in vivo* and its high-level expression when propionic acid metabolism is interrupted (25). However, it has become certain that both urine and the gut microbiome play crucial parts in the onset or prevention of kidney disease (26–28). When there is an excess of MMA in the body, the capacity to convert lactate to pyruvate is decreased, and thus MMA has an inhibitory effect on TCA cycling and gluconeogenesis-related effector enzymes (29). Impaired TCA cycling may also be associated with the regulation of inflammation and oxidative stress (30). In addition, MMA is involved in lipid metabolism and the urea cycle (31). TCA metabolites are considered by researchers to be important potential signals of renal impairment (32). Studies have shown that higher concentrations of MMA are nephrotoxic in inherited diseases of propionate metabolism, which might be expected (33). Therefore, MMA plays a role in promoting inflammatory damage and repair (34).

In addition, subgroup analyses revealed age, hypertension, and vitamin B_12_ levels as significant influences on the relationship between MMA and CKD. For age, older adults tend to exhibit higher levels of MMA, which may be attributed to age-related declines in renal function and changes in metabolism (35). On the one hand, the vascularity and function of the kidneys naturally deteriorate with age. This aging process may lead to a decline in the function of the renal tubules and interstitium, increasing the likelihood of MMA and CKD (36). On the other hand, as we age, there may be an increase in co-morbidities, such as diabetes and cardiovascular complications, which may increase the risk of CKD (37). In the case of hypertension, this may be due to the interaction between hypertension and worsening renal function, with chronic hypertension exacerbating renal damage and thus affecting MMA metabolism. On the one hand, hypertension can lead to damage and hardening of the small renal arteries, affecting the blood supply to the kidneys and thus aggravating the development of CKD (38). On the other hand, hypertension can lead to a decrease in glomerular filtration rate, which in turn impairs glomerular and tubular function and increases the risk of CKD (39). These findings offer valuable insights into the complexity of the MMA-CKD association and its potential modifiers.

In summary, we speculate that the accumulated MMA can participate in the formation of CKD through complex inflammatory injury, varieties of complex metabolic pathways, and signaling. This also provides a novel perspective for the prevention and treatment of CKD. However, how serum MMA affects the physiology of CKD patients has not been fully elucidated. We hypothesize that serum MMA may be involved in the pathogenesis of CKD, but the exact mechanism needs to be further investigated.

### 4.1 Study limitations and strengths

The research presents plenty of strengths to consider. To start with, the observation was quite large. Second, we adjusted for numerous confounding factors through multiple regression analysis to avoid bias to produce more reliable results. In addition, we performed stratified analyses to further explore its relationship in different subgroups. Moreover, this study reveals the relationship between MMA and the risk of CKD dose-response and finds a specific “W”-shaped relationship. The study examined the association between MMA and different stages of CKD. However, because of a few limitations, care should be taken when interpreting the study’s findings. First, discussing a causal relationship between them was not suitable for the cross-sectional design of this study (40). Second, variables gathered using questionnaires might not be correctly categorized. Third, the test methods and testing procedures for serum MMA may be biased, which may result in less accurate exposure variables. Fourth, other uncorrected confounders might exist. Therefore, further prospective studies with larger sample sizes or multicenter cohort studies need to be designed in this area in the future. Confirming these findings may involve incorporating more possible confounders, using standardized measurement methods, and ensuring a high degree of accuracy in the testing process.

## 5 Conclusions

In this sizable cross-sectional study with a population basis, 5,232 patients aged 20 years and older with CKD were included. Our study reveals that serum MMA increases as the stage of CKD progresses. Serum MMA is positively associated with the risk of CKD. Serum MMA may be a novel index to predict the development and course of CKD. This study may help in the early identification of people at risk for chronic kidney disease and provide new ideas and approaches for prevention and treatment.

## Data availability statement

Publicly available datasets were analyzed in this study. This data can be found here: https://wwwn.cdc.gov/nchs/nhanes/continuousnhanes/default.aspx?BeginYear=2013.

## Ethics statement

The studies involving humans were approved by National Center for Health Statistics Ethics Review Board. The studies were conducted in accordance with the local legislation and institutional requirements. The human samples used in this study were acquired from Serum samples are processed, stored, and shipped to the Division of Laboratory Sciences, National Center for Environmental Health, Centers for Disease Control and Prevention, Atlanta, GA for analysis. Detailed instructions on specimen collection and processing are discussed in the NHANES Laboratory Procedures Manual (LPM). Vials are stored under appropriate frozen (–30°C) conditions until they are shipped to National Center for Environmental Health for testing. For more information, see https://wwwn.cdc.gov/Nchs/Nhanes/2013-2014/MMA_H.htm. Written informed consent for participation was not required from the participants or the participants’ legal guardians/next of kin in accordance with the national legislation and institutional requirements.

## Author contributions

ZZ: Formal analysis, Investigation, Software, Visualization, Writing – original draft. LL: Formal analysis, Investigation, Software, Writing – original draft. SG: Investigation, Writing – original draft. FJ: Formal analysis, Supervision, Writing – original draft. DH: Supervision, Visualization, Writing – original draft. HS: Formal analysis, Visualization, Writing – original draft. WS: Funding acquisition, Supervision, Writing – review & editing. SJ: Writing – original draft, Writing – review & editing. FT: Writing – original draft, Writing – review & editing.
