# Supplementary material for: Association between serum methylmalonic acid and chronic kidney disease in adults: a cross-sectional study from NHANES 2013-2014
Source: Front Endocrinol (Lausanne). 2024 Aug 1;15:1434299. doi: 10.3389/fendo.2024.1434299 (PMC11324440; doi:10.3389/fendo.2024.1434299)
Supplement: Supplementary file 1 [file Table_1.docx]

Supplementary Material

**Supplementary Tables.** Statistical information on ln MMA levels in different CKD stages and results of the Kruskal-Wallis test.

| CKD Stage | Sample Count | Mean | SD | Median | 25th Percentile | 75th Percentile |
| --- | --- | --- | --- | --- | --- | --- |
| 1 | 31 | 4.848483 | 0.454978 | 4.875197 | 4.553249 | 5.117922 |
| 2 | 19 | 5.319556 | 0.545821 | 5.26269 | 4.90782 | 5.655894 |
| 3 | 35 | 5.589534 | 0.633365 | 5.433722 | 5.241523 | 5.708769 |
| 4 | 15 | 5.803086 | 0.780075 | 5.860786 | 5.600188 | 6.313074 |
| 5 | 7 | 6.649299 | 0.942315 | 6.375025 | 6.063515 | 6.771378 |
|  |  |  |  |  |  |  |
| K-W test results |  |  | H=50.47 | p-value: 2.88e-10 | |  |

These results clearly show that the median and range of distribution of ln MMA levels tend to increase with increasing CKD stage, especially at higher CKD stages. The results of the K-W test (p-value = 2.88e-10) strongly suggest that there is a significant difference in the distribution of ln MMA levels across CKD stages. This p-value is much smaller than any common level of significance (e.g., 0.05 or 0.01), indicating that we can reject the original hypothesis of no difference between groups. In conclusion, the results of these analyses further confirm that there is a positive correlation between MMA levels and CKD stage and that this relationship is statistically significant. As the CKD stage increased, MMA levels also increased significantly, especially at higher CKD stages. These findings emphasize the importance of monitoring and assessing MMA levels in CKD management and research.
